# Highly Prevalent Multidrug-Resistant *Campylobacter* spp. Isolated From a Yellow-Feathered Broiler Slaughterhouse in South China

**DOI:** 10.3389/fmicb.2021.682741

**Published:** 2021-06-16

**Authors:** Jie Bai, Zhengquan Chen, Kaijian Luo, Fanliang Zeng, Xiaoyun Qu, Hongxia Zhang, Kaifeng Chen, Qijie Lin, Haishan He, Ming Liao, Jianmin Zhang

**Affiliations:** ^1^Key Laboratory of Zoonoses, Ministry of Agriculture, Key Laboratory of Zoonoses Prevention and Control of Guangdong Province, Guangdong Laboratory for Lingnan Modern Agriculture, National and Regional Joint Engineering Laboratory for Medicament of Zoonoses Prevention and Control, College of Veterinary Medicine, South China Agricultural University, Guangzhou, China; ^2^Key Laboratory of Livestock Disease Prevention of Guangdong Province, Scientific Observation and Experiment Station of Veterinary Drugs and Diagnostic Techniques of Guangdong Province, Ministry of Agriculture, Institute of Animal Health, Guangdong Academy of Agricultural Sciences, Guangzhou, China

**Keywords:** multidrug-resistant *Campylobacter*, yellow broiler, slaughtering line, virulence genes, pulse field gel electrophoresis

## Abstract

The purpose of this study was to investigate the prevalence, antimicrobial resistance, virulence genes, and genetic diversity of *Campylobacter* spp. along the yellow-feathered broiler slaughtering line in Southern China from December 2018 to June 2019. A total of 157 *Campylobacter* spp. isolates were identified from 1,102 samples (including 53.6% (75/140) of live chicken anal swab samples, 27.5% (44/160) of defeathering samples, 18.1% (29/160) of evisceration samples, 2.1% (3/140) of washing samples, 1.4% (2/140) of chilling samples, and 1.1% (4/362) of environmental samples). The prevalence of *Campylobacter* spp. was 14.2%, including 43.9% *Campylobacter jejuni*, 53.5% *Campylobacter coli*, and 2.5% other *Campylobacter* species. The highest antimicrobial resistance rate was found to be against sulfamethoxazole (138/157, 87.9%), and 90.4% (142/157) of the isolates were multidrug resistant (MDR). Examination of resistance-related genes revealed the double base mutated Thr-86-Ile, which informed ACA-TTA, with an Arg-79-Lys substitution in *gyrA*. Eleven virulence-associated genes (*cadF*, *cdtA*, *cdtB*, *ciaB*, *flaA*, *imaA*, *dnaJ*, *plaA*, *virB11*, *racR*, and *cdtC*) were also detected by a polymerase chain reaction (PCR) analysis, and *cadF* (81.5%) was the most prevalent. Based on an analysis of pulsed-field gel electrophoresis (PFGE) results, we found that *Campylobacter* spp. could be cross-contaminated throughout the entire slaughtering line. These results show that it is imperative to study the *Campylobacter* spp. from the yellow-feathered broiler along the slaughtering line in China to develop preventative and treatment measures for the poultry industry, as well as food safety and public health.

## Introduction

*Campylobacter* spp. is the most common causative agent of foodborne diseases, with *Campylobacter jejuni* and *Campylobacter coli* representing (Newell and Fearnley, 2003; Ma et al., 2014). The United States Centers for Disease Control and Prevention (CDC) estimates that *Campylobacter* spp. infections affect more than 1.5 million people in the United States every year, moreover, there are additional cases that go undiagnosed or unreported (FoodNet and CDC, 2017). The National Institute of Nutrition and Food Safety of the Chinese Center for Disease Control and Prevention tested 879 raw poultry meat in 2007. The detection rate of *C. jejuni* was 1.82%, while the figure rose to 2.28% in 2008. More importantly, in rare cases, *Campylobacter* spp. can cause a serious complication known as Guillain-Barre syndrome, which is associated with a mortality rate as high as 3–10%; thus, monitoring the prevalence of *Campylobacter* spp. is necessary.

Broiler chickens intended for human consumption represent the primary mode of *Campylobacter* spp. transmission (Moore et al., 2005; Greige et al., 2019). Although yellow- and white-feathered broiler are two of the main types of broiler, current studies on *Campylobacter* spp. have focused mainly on white-feathered broiler. In China, yellow-feathered broiler is a Chinese-specific broiler industry. The production (head units) of live yellow-feathered broiler breeding was approximately 4.0 billion in 2016, which was comparable with the production of white-feathered broiler (Wang et al., 2019). In addition, slaughtering is a critical part of the “farm to fork,” and the scalding, chilling, defeathering, and evisceration processes represent sites of major cross-contamination and are of critical importance (Figueroa et al., 2009). As such, the European Commission has established microbiological processing hygiene criteria for *Campylobacter* spp. in broiler carcasses (European Commision, 2017); however, few studies have focused on the entire slaughtering chain of yellow-feathered broiler. Therefore, detecting the prevalence of *Campylobacter* spp. during the slaughtering process of yellow-feathered broiler is essential.

There is severe worldwide antimicrobial resistance of *Campylobacter* spp. in white-feathered broiler, especially multidrug-resistant (MDR) strains (Zbrun et al., 2015), including in China. In particular, resistance to fluoroquinolones [e.g., ciprofloxacin (CIP) and nalidixic acid (NAL)] is extremely high in some regions (Zhang et al., 2018). Furthermore, since macrolides (e.g., erythromycin) are common first-line treatments, the resistance to the macrolides in China is considerably higher than other countries (Chen et al., 2010; Bolinger and Kathariou, 2017); however, the long feeding cycle of yellow-feathered broiler may increase antibiotic use. Unfortunately, there are no previous studies that have assessed the resistance bacteria isolated from yellow-feathered broiler.

Thus, the aim of the present study was to elucidate the prevalence, antimicrobial resistance, virulence genes, and genetic diversity of *Campylobacter* spp. along the yellow-feathered broiler slaughtering line in Southern China. These findings provide a foundation of follow-up studies on risk assessment and food safety monitoring associated with *Campylobacter* spp.

## Materials and Methods

### Sample Collection

From December 2018 and June 2019, a total of 1,102 samples were collected from different stages of the slaughtering line (including defeathering, evisceration, washing, chilling, and live chicken anal swab samples) and the environment in a yellow-feathered broiler slaughterhouse in Guangdong province, China. The description of the number and type of samples are listed in Table 1. The specific sampling methods for each of the different links are based on previously described methods (Han et al., 2019). Each sample was labeled, transferred to the laboratory within 2 h, and processed immediately.

**TABLE 1:** Prevalence of *Campylobacter* spp. in the yellow-feathered broiler slaughterhouse.

Source	Prevalence of 157 *Campylobacter* spp.				Total
	Sample	*C. jejuni*	*C. coli*	Other species	
Slaughtering line					
Live chicken anal swab	140	33 (23.6%)	42 (30%)	–	75 (53.6%)
Defeathering (carcass)	160	22 (13.8%)	22 (13.8%)	–	44 (27.5%)
Evisceration (carcass)	160	9 (5.6%)	20 (12.5%)	–	29 (18.1%)
Washing (carcass)	140	3 (2.1%)	–	–	3 (2.1%)
Chilling (carcass)	140	2 (1.4%)	–	–	2 (1.4%)
Environmental					
Evisceration (water)	80	–	–	1 (1.3%)	4 (1.1%)
Washing (water)	80	–	–	–	
Sterilizing water	42	–	–	–	
Defeathering (water)	80	–	–	–	
Ground (swab)	40	–	–	2 (5.0%)	
3-Pronged hook (swab)	40	–	–	1 (2.5%)	
Total	1,102	69 (43.9%)	84 (53.5%)	4 (2.5%)	

*“–” Means no *Campylobacter* spp. was detected.*

### *Campylobacter* spp. Isolation and Identification

*Campylobacter* spp. isolation and identification was performed according to the Standard ISO 10272-1: 2006 (International Organization for Standardization, 2006) method (Han et al., 2016, 2019). For the live chicken anal swab samples, Skirrow blood agar containing 5% defibrinated sheep blood was incubated at 42°C for 36–48 h under a microaerophilic conditions (85% N_2_, 10% CO_2_, 5% O_2_). The poultry carcass samples were subjected to a broth culture in a 50-ml centrifuge tube, after which 1 ml of enrichment was added to 9 ml Bolton broth (with 5% defibrinated sheep blood), and then incubated at 42°C for 48 h under the same conditions. For the environmental swabs and water samples, the suspension was cultivated at 37°C in a shaker at 100 rpm for 2–4 h. Next, 1 ml of enrichment was added to 9 ml Bolton broth containing 5% defibrinated sheep blood and incubated at 42°C for 48 h under the same conditions.

Smooth, translucent, drop-shaped suspected colonies on the selective culture medium were selected and identified using a series of methods, including Gram staining and biochemical testing (production of catalase, oxidase test, growth test, hippurate hydrolysis, indoxyl acetate hydrolysis, and susceptibility to cephalotin). The presumptive isolates underwent further confirmation by multiplex PCR targeting of the *16S rDNA* gene of *Campylobacter* spp., *MapA* gene of *C. jejuni*, and *ceuE* gene of *C. coli*. PCR was conducted with the primers listed in Supplementary Table 1.

### Antimicrobial Susceptibility Testing

Susceptibility against antibiotics was evaluated using the disk diffusion technique (K–B method), and the results were read based on the National Committee for Clinical Laboratory Standards (NCCLS). Twelve antibiotics belonging to nine different classes were tested (Supplementary Table 1). *Campylobacter* spp. isolates were tested for their susceptibility to CIP, NAL, gentamicin (GEN), clindamycin (CLI), tetracycline (TET), erythromycin (ERY), amikacin (AMK), streptomycin (STR), florfenicol (FFC), ampicillin (AMP), sulfamethoxazole (SXT), and tigecycline (TGC). *C. jejuni* (NCTC 11168) was set as the quality control. Isolates exhibiting resistance to three or more antibiotic classes were defined as MDR.

### Detection of Resistance and Virulence-Associated Genes

All *Campylobacter* spp. isolates were screened for the presence of resistance and virulence genes by PCR. The DNA templates were prepared according to a previously described method (Han et al., 2019). The primers used to amplify the resistance genes in this study are listed in Supplementary Table 2 and virulence-associated genes are shown in Supplementary Table 3. PCR products were analyzed by agarose gel electrophoresis (1%) and sent to Sangon Biotech Co., Ltd. (Shanghai, China) for sequencing. Sequence data were then analyzed by DNAstar (DNAstar Inc., Madison, WI, United States), and the sequences were aligned using GenBank online BLAST software^1^.

### Pulsed-Field Gel Electrophoresis

The isolates were subjected to molecular typing by pulsed-field gel electrophoresis (PFGE), which was performed using the PulseNet standardized protocol. PFGE was performed after digestion of the genomic DNA with the restriction enzyme, Sam I, *Salmonella enterica* subsp. *enterica* serovar Braenderup (CDC no. H9812), was used as the standard control strain. The PFGE results were analyzed using BioNumerics Software. A PFGE pattern was defined as a group of strains with a Dice coefficient similarity of 85% or higher and the PFGE pattern represented by multiple strains was a PFGE cluster.

### Statistical Analysis

A comparison of frequencies was calculated using a Fisher’s exact test with GraphPad Prism 7.0. A *P*-value < 0.05 was considered to indicate statistical significance.

## Results

### Prevalence of *Campylobacter* spp. in the Yellow-Feathered Broiler Slaughterhouse

**FIGURE 1:**
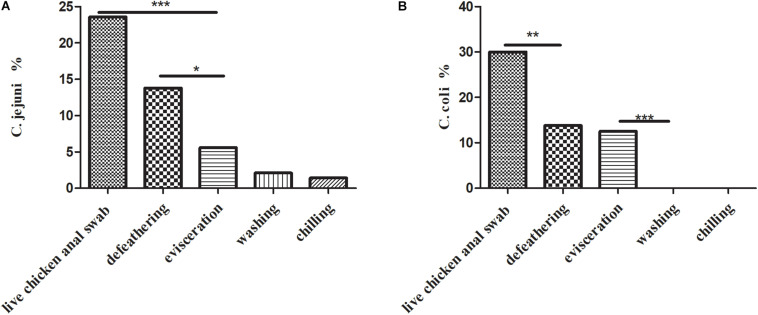
**(A)** Prevalence of *C. jejuni* isolates (*n* = 69) in the slaughtering line. A Fisher’s exact test was used for the categorical variables. ***P < 0.01, *P < 0.05. **(B)** Prevalence of *C. coli* isolates (*n* = 84) in the slaughtering line. A Fisher’s exact test was used for the categorical variables. ***P < 0.01, **P < 0.05.

A total of 157 (157/1102, 14.2%) *Campylobacter* spp. isolates were identified from 1,102 samples (Table 1), which consisted of a high prevalence in three processes: (1) 75 (75/140, 53.6%) isolates from live chicken anal swabs; (2) 44 (44/160, 27.5%) isolates from defeathering; and (3) 29 (29/160, 18.1%) isolates from evisceration. Moreover, 53.5% (84/157) of isolates were *C. coli*, which was the predominant factors in this study. Figure 1A presents the prevalence of *C. jejuni* from different sources. Two significant decreases were observed for *C. jejuni*: (1) from 23.6% (33/140) in the process of live chicken anal swabs to 13.8% (22/160) during defeathering and (2) 13.8% (22/160) during defeathering to 5.6% (9/140) in evisceration. In contrast, the positive rate of *C. coli* declined rapidly from 30% (42/140) in the live chicken anal swabs to 13.8% (22/160) defeathering, and the level of contamination remained at 12.5% (20/160) during evisceration, then fell to 0% during the washing and chilling processes (Figure 1B).

### Multidrug-Resistant *Campylobacter* spp. Isolates

**FIGURE 2:**
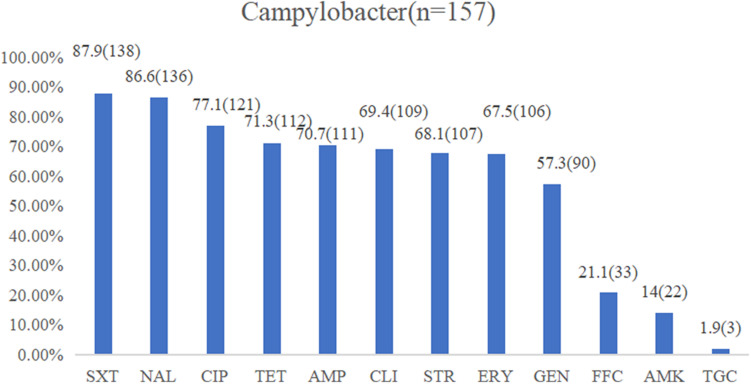
Resistance proportions of *Campylobacter* spp. from the yellow-feathered broiler slaughterhouse against 12 antibiotics. Abbreviations for antimicrobial agents: SXT, sulfamethoxazole; NAL, nalidixic acid; CIP, ciprofloxacin; TET, tetracycline; AMP, ampicillin; CLI, clindamycin; STR, streptomycin; ERY, erythromycin; GEN, gentamicin; FFC, florfenicol; AMK, amikacin; TGC, tigecycline.

The isolates exhibiting resistance to sulfamethoxazole (87.9%), nalidixic acid (86.6%), ciprofloxacin (77.1%), and tetracycline (71.3%) were commonly observed, followed by ampicillin (70.7%), clindamycin (69.4%), streptomycin (68.1%), erythromycin (67.5%), and gentamicin (57.3%) with a medium resistance level. Low resistance levels were observed in florfenicol (21.1%), amikacin (14%), and tigecycline (1.9%) (Figure 2). In total, 142 isolates (90.4%) were found to be resistant to at least three classes of antimicrobial agents, which were classified as MDR strains (Table 2). The multiple drug resistance rate for the washing and chilling processes was 100%, whereas the rates for the live chicken anal swab, defeathering, and evisceration displayed rates of 92, 88.6, and 86.2%, respectively. Analysis by species showed that, 89.9% of *C. jejuni*, 86.9% of *C. coli*, and 100% of other *Campylobacter* species were identified as MDR isolates.

**TABLE 2:** Multidrug-resistant (MDR) *Campylobacter* spp. isolates from different sources and species^1^.

Species	*A* (*n* = 75)	*B* (*n* = 44)	*C* (*n* = 29)	*D* (*n* = 3)	*E* (*n* = 2)	*F* (*n* = 4)	Total (%)
5 > *X* ≧ 3							
*C. jejuni*	5	7	3	0	0	0	33/142 (23.2)
*C. coli*	6	4	8	0	0	0	
Other	0	0	0	0	0	0	
7 > *X* ≧ 5							
*C. jejuni*	9	7	2	1	1	0	53/142 (37.3)
*C. coli*	14	12	4	0	0	0	
Other	0	0	0	0	0	3	
*X* ≧ 7							
*C. jejuni*	16	6	3	2	1	0	56/142 (39.4)
*C. coli*	19	3	5	0	0	0	
Other	0	0	0	0	0	1	
Total (%)	69/75 (92.0)	39/44 (88.6)	25/29 (86.2)	3/3 (100.0)	2/2 (100.0)	4/4 (100.0)	142/157 (90.4)

**C. jejuni* (*n* = 69) 62/69 (89.9); *C. coli* (*n* = 84) 73/84 (86.9); Other (*n* = 4) 4/4 (100.0). *X*, the number of antibiotic resistant classes; *C. jejuni*, *Campylobacter jejuni*; *C. coli*, *Campylobacter coli*; *A*, the source of live chicken anal swab; *B*, the source of defeathering; *C*, the source of evisceration; *D*, the source of washing; *E*, the source of chilling; *F*, the source of environmental.*

### Analysis of the Genes and Sequencing Associated With Antibiotic Resistance

**FIGURE 3:**
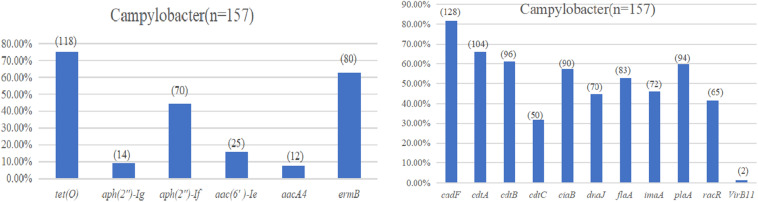
**(A)** Drug resistance gene carrying rate of *Campylobacter* spp. **(B)** Virulence determinants carrying rate of *Campylobacter* spp.

The test results of the amplification resistance genes are presented in Figure 3A. In general, 75.2% (118/157) of the isolates were positive for the carriage of the tetracycline-resistant gene, *tetO*. Sixty-three percent (80/157) of the isolates was identified as erythromycin-resistant gene, *ermB*, in the study. With regard to the aminoglycoside-resistant genes, the highest overall level of resistance gene was observed for *aph(2*″*)-Ig* at 44.6% (70/157), followed by *aac(6*′*)-Ie* at 15.9% (25/157), *aph(2*″*)-If* at 8.9% (14/157), and *aacA4* at 7.6% (12/157).

An alignment of the deduced amino acid consensus sequences of the isolates resistant to erythromycin and fluoroquinolone (FQ) with published sequences are presented in Table 3. Among the 106 erythromycin-resistant isolates, the C2113T (36.8%) and A2075G (18.9%) mutations in the *23S rRNA* gene were observed. Moreover, a series of point substitutions were identified in the *gyrA* gene from the 136 quinolone-resistant isolates. The Thr-86-Ile substitution (27.9%) was identified in the nalidixic acid resistance isolates, and 23.5% isolates of the nalidixic acid resistance isolates were found to possess a new double-base mutation in Thr-86-Ile with an Arg-79-Lys substitution. However, the other nalidixic acid resistance isolates did not display mutations.

**TABLE 3:** The mutations in antimicrobial resistance genes, *23S rRNA* and *gyrA.*

Gene	Mutations	Proportion (%)	Total (%)
*23S rRNA*	A2075G	20/106 (18.9)	59/106 (55.7)
	C2113T	39/106 (36.8)	
*gyrA*	ACA-ATA Thr-86-Ile	38/136 (27.9)	70/136 (51.5)
	ACA-TTA Thr-86-Ile with Arg-79-Lys	32/136 (23.5)	

### Prevalence and Distribution of *Campylobacter* spp. Virulence Determinants

The virulence determinant of *cadF* (81.5%) was the most prevalent in all the isolates, followed by *cdtA* (66.2%), *cdtB* (61.1%), *plaA* (59.9%), *ciaB* (57.3%), *flaA* (52.9%), *imaA* (45.9%), *dnaJ* (44.6%), *racR* (41.4%), and *cdtC* (31.8%). Only two isolates (1.3%) carried the *virB11* gene, which was located in a plasmid (Figure 3B). There were 96 (61.1%) strains that coharbored at least five virulence determinants. Among these isolates, 24 (25.0%) isolates that cocarried 10 virulence genes were dominant. Compared with different sources, the 22 (75.9%) strains isolated from the evisceration process were the highest, and species 57 (67.9%) of *C. coli* was predominant (Table 4).

**TABLE 4:** *Campylobacter* spp. isolates coharbored the number of virulence determinants in different sources and species.

The number of virulence determinants	Species	*A* (*n* = 75)	*B* (*n* = 44)	*C* (*n* = 29)	*D* (*n* = 3)	*E* (*n* = 2)	*F* (*n* = 4)	Total (%)
5	*C. jejuni*	2	1	0	1	0	0	14/96 (14.6)
	*C. coli*	4	3	3	0	0	0	
	Other	0	0	0	0	0	0	
6	*C. jejuni*	1	2	2	0	0	0	21/96 (21.9)
	*C. coli*	10	2	4	0	0	0	
	Other	0	0	0	0	0	0	
7	*C. jejuni*	1	5	1	2	1	0	12/96 (12.5)
	*C. coli*	2	1	0	0	0	0	
	Other	0	0	0	0	0	1	
8	*C. jejuni*	1	1	1	1	0	0	9/96 (9.4)
	*C. coli*	4	0	1	0	0	0	
	Other	0	0	0	0	0	0	
9	*C. jejuni*	3	0	1	0	0	0	16/96 (16.7)
	*C. coli*	3	4	4	0	0	0	
	Other	0	0	0	0	0	1	
10	*C. jejuni*	3	5	1	0	1	0	24/96 (25)
	*C. coli*	3	5	4	0	0	0	
	Other	0	0	0	0	0	2	
Total (%)		37/75 (49.3)	29/44 (65.9)	22/29 (75.9)	2/3 (66.7)	2/2 (100)	4/4 (100)	96/157 (61.1)

**C. jejuni* (*n* = 69) 35/69 (50.7); *C. coli* (*n* = 84) 57/84 (67.9); Other (*n* = 4) 4/4 (100). *C. jejuni*, *Campylobacter jejuni*; *C. coli*, *Campylobacter coli*; *A*, the source of live chicken anal swab; *B*, the source of defeathering; *C*, the source of evisceration; *D*, means the source of washing; *E*, the source of chilling; *F*, the source of environmental.*

### Pulsed-Field Gel Electrophoresis

A total of 69 *C. jejuni* and 84 *C. coli* isolates, representing isolates of different sources and species were selected for PFGE analysis after digestion by *Sma*I. Consequently, three isolates from *C. jejuni* and one from *C. coli* were subjected to three repeated trials, and genotypes could not be identified by PFGE.

**FIGURE 4:**
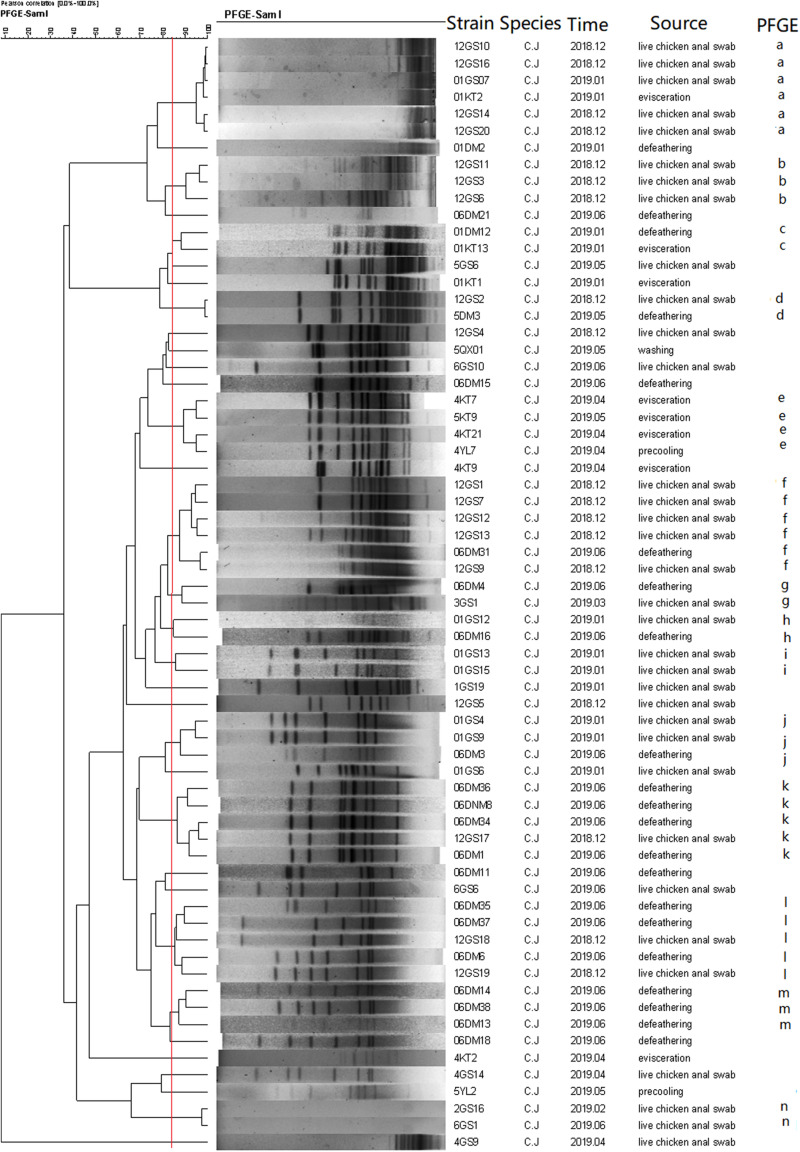
Dendrogram of *Sma*I PFGE patterns of 66 *C. jejuni* isolates from five stages of the chicken slaughtering chain. (a–n) PFGE patterns.

**FIGURE 5:**
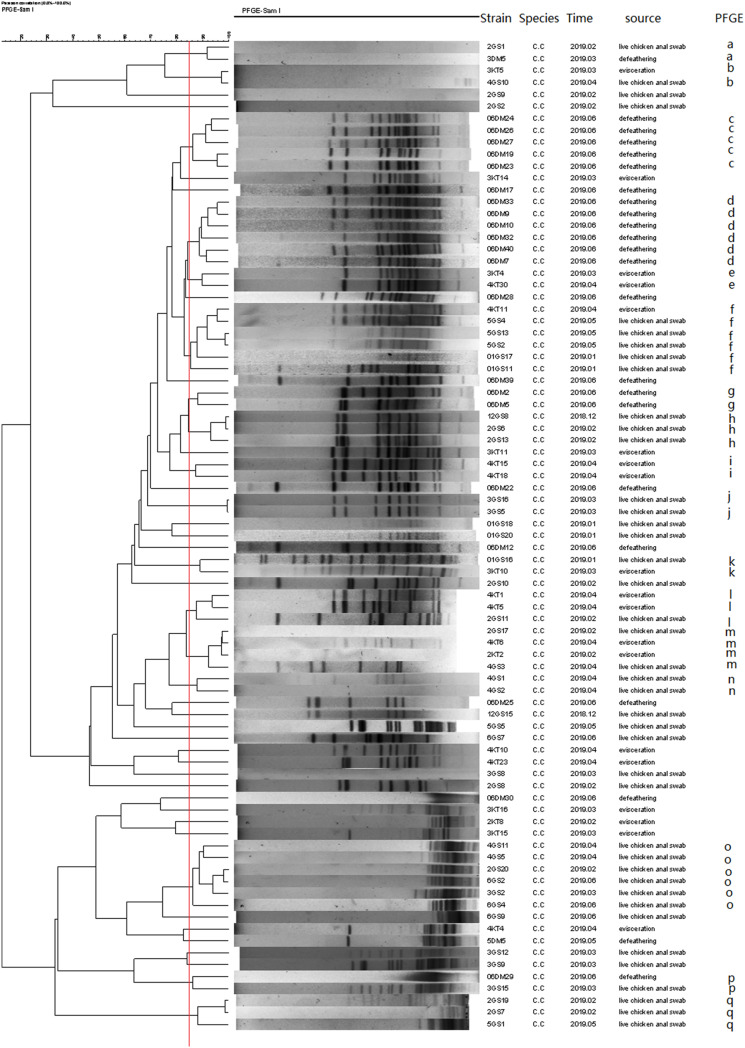
Dendrogram of the *Sma*I PFGE patterns of 83 *C. coli* isolates from five stages of the chicken slaughtering chain. (a–q) PFGE patterns.

As a result, the 66 *C. jejuni* isolates were grouped into 14 clusters (a–n) (Figure 4), represented by multiple strains, and 19 unique PFGE patterns, represented by a single strain. The 83 *C. coli* isolates were grouped into 17 clusters (Figure 5) and 29 unique PFGE patterns. The *C. jejuni* isolates were dominant in cluster f, which included five isolates from the swab samples and one from the defeathering samples. Furthermore, the *C. coli* isolates had three dominant clusters of d, f, and o. In cluster d, all six isolates were derived from the defeathering samples. In cluster f, five isolates were derived from live chicken anal swab samples and one evisceration sample. In cluster o, all six isolates were derived from live chicken anal swab samples. In terms of the slaughtering line, the live chicken anal swab samples carried the most PFGE patterns (34 patterns), followed by defeathering samples (24 patterns), evisceration samples (19 patterns), chilling (2 patterns), and washing (1 patterns). The isolates that belonged to the same genotype could be recovered from different origins (i.e., the *C. jejuni* isolates in clusters d and f). In addition, the isolates from one source could be identified in the same genotype (i.e., the *C. coli* isolates in cluster o).

## Discussion

*Campylobacter* spp. play a significant role in the food safety. Yellow-feathered broiler is Chinese local dominant species, which exhibits obvious regional characteristics associated with production and consumption distribution. Guangdong is the largest yellow-feathered broiler-producing province in China, and one of the main consumer regions. Compared with the white broiler, the yellow-feathered broiler has a longer growth cycle, and the chilling is often performed at the final stage of the yellow-feathered broiler slaughter and sold as whole chickens. In contrast, while the white-feathered broiler is subjected to the segmentation process and often sold by dividing the animal into different parts. As such, these disparities in processing will have different impacts on consumer food safety issues. However, few studies have investigated the contamination of *Campylobacter* spp. in yellow-feathered broiler slaughterhouses. This is the first study with a research focus on the whole yellow-feathered broiler slaughtering chain in China.

The separation rate of *Campylobacter* spp. from the slaughterhouse reached a low point of 14.2% in this study, compared with previous reports from white-feathered broiler or turkey slaughterhouse ranging from 26.3 to 100% in China and some other Asian and African countries (Chen et al., 2010; Ma et al., 2014; Messad et al., 2014; Kojima et al., 2015; Han et al., 2016). The low separation rate that was obtained may be due to undivided slaughter with the use of disinfectants to reduce the level of cross-contamination or the sample collection methods, seasonal factors (Zendehbad et al., 2015), and the strict biosecurity measures used by the slaughterhouse (Sasaki et al., 2014), may also account for their viable but non-culturable (VBNC) state. Thus, under adverse conditions, *Campylobacter* spp. may enter a VBNC state.

The prevalence of *Campylobacter* spp. varied between the different sources in this study. In the entire slaughtering chain, a gradual downward trend was observed from the live chicken to the finished product, which was similar to a study of white-feathered broiler in Iran; however, the positive rate of every aspect was not found to be as high as reported in that study (Rahimi et al., 2010). This difference may be caused by the use of the entire chicken as a finished product to reduce the body surface area exposed to the processing water in the scalder and chiller tanks. These have often been considered to be sources of cross-contamination on carcasses that potentially affect the microbial profile of the final product (Munther et al., 2016). Our results indicate that the defeathering and evisceration processes were the key factors required to control the contamination in the slaughter chain, which is consistent with previous reports (Huang et al., 2018). Among the isolates, *C. coli* was the predominant species in our study, which accounted for 53.5% of *Campylobacter* spp., which differed from that of previous reports (Chen et al., 2010; Melero et al., 2012; Kittl et al., 2013).

The *Campylobacter* spp. isolates displayed substantial drug resistance. The fluoroquinolones were found to exhibit a high resistance rate, which was in accordance with that of previous reports (Hungaro et al., 2014; Zbrun et al., 2015; Panzenhagen et al., 2016). Sulfonamide and tetracycline were maintained at high levels, which was a predictable result for its unreasonable use in the chicken industry; however, a relatively high resistance was observed for the first-line drugs, erythromycin (67.5%) and gentamicin (57.3%) compared with research conducted in Poland, Japan, Tranidala island, and Algeria (Rodrigo et al., 2007; Sallam, 2007; Maćkiw et al., 2012; Messad et al., 2014). Coincidentally, it remained in a similar level with recent reports in China (Han et al., 2016, 2019; Li et al., 2017). Furthermore, one serious drug resistance strain was discovered in the present study, which means it was resistant to all 12 of the tested antibiotics, including tigecycline, which represented an alarming state of affairs.

In total, 90.4% were classified as MDR strains, which was substantially higher than reported in previous research (Rodrigo et al., 2007; Sallam, 2007; Zbrun et al., 2015; Zendehbad et al., 2015). Moreover, the MDR rates were higher than 80% for the processes of the live chicken anal swab, defeathering, and evisceration, while the rate was 100% for the washing and chilling processes. In addition, 89.9% of *C. jejuni*, 86.9% of *C. coli*, and 100% of other *Campylobacter* spp. were identified as MDR isolates. Due to the long feeding cycle of yellow-feathered broiler, there may be increased opportunities for the yellow-feathered broiler meat to obtain antibiotic resistance to *Campylobacter* spp. It appears that the drug-resistant situation in China is critical, which may be caused by the widespread use of antimicrobial agents during the breeding process of poultry and livestock. Therefore, in order to obtain antibiotic-free meat, the promotion and implementation of antibiotic-free breeding regarding the use of physical and biological measures of animal health and disease prevention without any chemical drugs, antibiotics, or synthetic hormones during the breeding or slaughtering process is considered.

The macrolide resistance gene, *23S rRNA*, and fluoroquinolone resistance gene, *gyrA*, were sequenced to analyze their mutations. In total, 36.8% of erythromycin-resistant strains possessed C2113T mutation, which was higher than A2075G mutation (18.9%) (Pérez-Boto et al., 2014; Lim et al., 2017). A 51.5% (70/136) Thr-86-Ile substitution was found in the tested isolates, as the common single base mutation, ACA-ATA (Ge et al., 2005; Abd El-Tawab et al., 2018). However, a new double-base mutation in Thr-86-Ile was detected in 32 isolates, which was termed ACA-TTA. Concurrent with the Arg-79-Lys substitution, other nalidixic acid resistance isolates did not display any type of mutation. This finding implies that the strains were likely to find a new method of survival and spread in extreme environments.

*Campylobacter* spp. has complex multifactorial systems for multiplication in broilers, survival during food processing, and enhanced pathogenicity following food processing stressors (Bolton, 2015). A total of 11 virulence determinants were detected, of which 61.7% of the strains coharbored at least five virulence determinants. Most of the isolates carried 10 virulence genes, accounting for 25%. There were only two isolates that carried the *virB11* gene, which can significantly reduce adherence and invasion compared with the wild-type strain (Bacon et al., 2000). More importantly, 75.9% strains from the evisceration stage and 67.9% of *C. coli* cocarried at least five virulence determinants. This finding indicates that the isolates from the evisceration stage and *C. coli* exhibited strong potential pathogenicity.

In this study, multiple PFGE patterns and clusters were observed in the *C. jejuni* and *C. coli* isolates, which indicated that the genome was polymorphic. Furthermore, the *C. coli* isolates had more PFGE patterns (29) than the *C. jejuni* isolates, which had 19 PFGE patterns. This suggests that the genes of the *C. coli* isolates are more unstable and variable than that of the *C. jejuni* isolates. Most PFGE clusters correspond to only one origin; however, the isolates that belonged to the same genotype could be recovered from different origins (Ma et al., 2014). This revealed that *Campylobacter* spp. could be cross-contaminated throughout the entire slaughtering line and might have serious consequences for the prevention in the poultry industry.

## Conclusion

In summary, this represents the first attempt to gather information of *Campylobacter* spp. from a yellow-feathered broiler slaughterhouse in China. Moreover, we showed a significant reduction of *C. jejuni*- and *C. coli*-positive isolates during the process of defeathering and evisceration, serious MDR in *Campylobacter* spp., and novel mutation in the *gyrA* genes. The PFGE results implied that serious cross-contamination occurred in the slaughtering line, which requires future focus in order to reduce the level of *Campylobacter* spp. from the slaughterhouse to retail outlets. Furthermore, we should restrict the use of antibiotics in livestock and implement monitoring to control the food safety of high risk food products.

## Data Availability Statement

The original contributions presented in the study are included in the article/Supplementary Material, further inquiries can be directed to the corresponding author/s.

## Author Contributions

JB: methodology, data curation, and writing (original draft preparation). ZC: validation and investigation. KL: supervision and resources. FZ: validation. XQ, HZ, KC, QL, and HH: investigation. ML: supervision and project administration. JZ: conceptualization and writing (reviewing and editing). All authors contributed to the article and approved the submitted version.

## Conflict of Interest

The authors declare that the research was conducted in the absence of any commercial or financial relationships that could be construed as a potential conflict of interest.

## Footnotes

1 http://www.ncbi.nlm.nih.gov/BLAST/
