# Supplementary material for: Highly Prevalent Multidrug-Resistant Campylobacter spp. Isolated From a Yellow-Feathered Broiler Slaughterhouse in South China
Source: Front Microbiol. 2021 Jun 16;12:682741. doi: 10.3389/fmicb.2021.682741 (PMC8242590; doi:10.3389/fmicb.2021.682741)
Supplement: Supplementary file 1 [file Data_Sheet_1.docx]

**SUPPLEMENTARY TABLE S1 |** PCR primers used to identify *Campylobacter* spp.

| **Genes** | **Primers sequence (5′–3′)** | **TM (°C)** | **Product size (bp)** | **References** |
| --- | --- | --- | --- | --- |
| *16S rRNA* (*Campylobacter*) | 5′-ATCTAATGGCTTAACCATTAAAC-3′ | 59 | 857 | Denis et al. (1999) |
|  | 5′-GGACGGTAACTAGTTTAGTATT-3′ |  |  |  |
| *MapA* (*C. jejuni*) | 5′-CTATTTTATTTTTGAGTGCTTGTG-3′ | 59 | 589 | Denis et al. (1999) |
|  | 5′-GCTTTATTTGCCATTTGTTTTATTA-3′ |  |  |  |
| *CeuE* (*C. coli*) | 5′-AATTGAAAAATTGCTCCAACTATG-3′ | 59 | 462 | Denis et al. (1999) |
|  | 5′-TGATTTTATTATTTGTAGCAGCG-3′ |  |  |  |

**SUPPLEMENTARY TABLE S2** | PCR primers used to identify drug resistance genes.

| Genes | Primers sequence (5′–3′) | TM (℃) | Product size (bp) | Reference |
| --- | --- | --- | --- | --- |
| *tet(O)* | 5′-GCGTTTTGTTTATGTGCG-3′ | 54 | 559 | Obeng et al. (2012) |
|  | 5′-ATGGACAACCC GACAGAAG-3′ |  |  |  |
| *23S rRNA* | 5′-GCTCGAAGGTTAATTGATG-3′ | 57 | 852 | Pérez-Boto et al. (2014) |
|  | 5′-GCTCTTGGCAGAACAAC-3′ |  |  |  |
| *L22* | 5′-TTAGCTTTCCTTTTTCACTGTTGCTTT-3′ | 55 | 425 | Corcoran, Quinn, Cotter, and Fanning (2006) |
|  | 5′-ATGAGTAAAGCATTA ATTAAATTCATAAG-3′ |  |  |  |
| *L4* | 5′-TTATCCCTCTTTTGTAATAGATTCTAA-3′ | 51 | 614 | Corcoran et al. (2006) |
|  | 5′-ATGAGTAAAGTAGTTGTTTTA AATGAT-3′ |  |  |  |
| *ermB* | 5′-CAGGTAAAGGGCATTTAACGACG-3′ | 58 | 436 | Zhou et al. (2016) |
|  | 5′-CATCTGTGGTATGGCGGGTAAG-3′ |  |  |  |
| *aph2-Ig* | 5′-TTGAGAATGCCATGGAACGGATCGG-3′ | 60 | 864 | Zhao et al. (2015) |
|  | 5′-GGGTCTATGGCTAGAAGTTCACGG-3′ |  |  |  |
| *aph(2")-If* | 5′-AAGGAACTTTTTTAACACCAG-3′ | 50 | 420 | Zhao et al. (2015) |
|  | 5′-CCWATTTCTTCTTCACTATCTTC-3′ |  |  |  |
| *aac(6′)-Ie* | 5′-ACAGAGCCTTGGGAAGATGAAG-3′ | 54 | 1,106 | Zhao et al. (2015) |
|  | 5′-TGTTCCTATTTCTTCTTCACTATC-3′ |  |  |  |
| *aacA4* | 5′-ATCTCATATCGTCGAGTGGAC-3′ | 50 | 440 | Zhao et al. (2015) |
|  | 5′-CGTGTTTGA ACCATGTAC-3′ |  |  |  |
| *gyrA* | 5′-CAACTGGTTCTAGCCTTTTG-3′ | 55 | 1,081 | Luo et al. (2005) |
|  | 5′-AATTTCACTCATAGCCTCACG-3′ |  |  |  |

**SUPPLEMENTARY TABLE S3** | PCR primers used to identify virulence genes.

| Genes | Primers sequence (5′–3′) | TM (℃) | Product size (bp) | Reference |
| --- | --- | --- | --- | --- |
| *flaA* | 5′-ATGGGATTTCGTATTAACAC-3′ | 45 | 1,713 | Wassenaar and Newell (2000) |
|  | 5′-CTGTAGTAATCTTAAAACATTTTG-3′ |  |  |  |
| *iamA* | 5′-GCACAAAATATATCATTACAA-3′ | 52 | 518 | Müller, Schulze, Müller, and Hänel (2006) |
|  | 5′-TTCACGACTACTATGAGG-3′ |  |  |  |
| *virB11* | 5′-GAACAGGAAGTGGAAAAACTAGC-3′ | 50 | 708 | Bacon et al. (2000b) |
|  | 5′-TTCCGCATTGGGCTATATG-3′ |  |  |  |
| *cadF* | 5′-TTGAAGGTAATTTAGATATG-3′ | 45 | 400 | Konkel, Gray, Kim, Garvis, and Yoon (1999) |
|  | 5′-CTAATACCTAAAGTTGAAAC-3′ |  |  |  |
| *ciaB* | 5′-TGCGAGATTTTTCGAGAATG-3′ | 45 | 527 | Ganan et al. (2010) |
|  | 5′-TGCCCGCCTTAGAACTTACA-3′ |  |  |  |
| *cdtA* | 5′-GGAAATTGGATTTGGGGCTATACT-3′ | 50 | 165 | Bang et al. (2003) |
|  | 5′-ATCACAAGGATAATGGACAAT-3′ |  |  |  |
| *cdtB* | 5′-GTTGGCACTTGGAATTTGCAAGGC-3′ | 50 | 495 | Bang et al. (2003) |
|  | 5′-GTTAAAATCCCCTGCTATCAACCA-3′ |  |  |  |
| *cdtC* | 5′-ATTACTTTGTTTTTTATGTTT-3′ | 50 | 237 | Müller et al. (2006) |
|  | 5′-TTGCACATAACCAAAAGGAAG-3′ |  |  |  |
| *dnaJ* | 5′-AAGGCTTTGGCTCATC-3′ | 46 | 720 | Datta (2003) |
|  | 5′-CTTTTTGTTCATCGTT-3′ |  |  |  |
| *racR* | 5′-GATGATCCTGACTTTG-3′ | 45 | 584 | Datta (2003) |
|  | 5′-TCTCCTATTTTTACCC-3′ |  |  |  |
